# Optimal Gating Window for Respiratory-Gated Radiotherapy with Real-Time Position Management and Respiration Guiding System for Liver Cancer Treatment

**DOI:** 10.1038/s41598-019-40858-2

**Published:** 2019-03-13

**Authors:** Se An Oh, Ji Woon Yea, Sung Kyu Kim, Jae Won Park

**Affiliations:** 1 Department of Radiation Oncology, Yeungnam University Medical Center, Daegu, Korea; 2 Department of Radiation Oncology, Yeungnam University College of Medicine, Daegu, Korea

## Abstract

Respiratory-gated radiotherapy is one of the most effective approaches to minimise radiation dose delivery to normal tissue and maximise delivery to tumours under patient’s motion caused by respiration. We propose a respiration guiding system based on real-time position management with suitable gating window for respiratory-gated radiotherapy applied to liver cancer. Between August 2016 and February 2018, 52 patients with liver cancer received training in real-time position management and respiration guiding. Respiration signals were statistically analysed during unguided respiration and when applying the respiration guiding system. Phases of 30–60% and 30–70% retrieved the lowest respiration variability among patients, and 47 patients exhibited significant differences in terms of respiration reproducibility between unguided and guided respiration. The results suggest that either of these phases can establish suitable windows for gated radiotherapy applied to liver cancer, especially regarding respiration reproducibility.

## Introduction

Several studies have recently addressed the minimisation of radiation delivered to normal tissue while focusing the radiation dose to tumours during radiotherapy under patient’s respiration motion, which is especially evident in regions such as liver and lungs. For instance, the Task Group 76^1^ from the American Association of Physicists in Medicine addressed radiotherapy in areas surrounding thoracic, abdominal, and pelvic tumours, which might be affected by respiratory motion. Specifically, they addressed the magnitude of respiratory motion, related problems, techniques to regulate respiratory motion and their application to patient care, including quality assurance guidelines during radiotherapy. Furthermore, their report explains the latest techniques applicable to tumour sites that tend to move with respiration. These techniques include motion encompassing^2–6^, respiratory gating^7–10^, breath holding^11–15^, forced shallow-breathing methods^16–19^, and respiratory synchronization^20^.

Several aspects affect real-time tumour-tracking radiotherapy, including the correlation between external marker, internal marker, and tumour. In clinical practice, the application of these treatments assumes a strong correlation between the locations of the external marker and tumour^21,22^. Beddar *et al*.^21^ evaluated eight patients who had undergone respiratory-based computed tomography (CT) during liver cancer radiotherapy. The implanted internal fiducial markers were gold seeds, whereas the external fiducial markers were those used with a real-time position management (RPM) system for respiratory gating. Regarding correlation between internal and external markers, the gating window corresponding to respiration phase 40–60% (i.e. percentage of a respiration cycle) produces the lowest variability during fiducial marker motion predominantly in the superior–inferior direction. Gierga *et al*.^22^ obtained similar motion correlations between an internal radiopaque tumour fiducial clip and an external marker in four patients with abdominal tumours using fluoroscopy and computer vision.

Nevertheless, the best respiration phase to obtain suitable gating windows remains to be determined for respiratory-gated radiotherapy (RGRT) of liver cancer. If the gating window is excessively short, the treatment time can be very long and may discomfort the patient. Otherwise, a very long gating window can render RGRT ineffective. The best gating window depends on the respiration motion from the abdomen over the liver region of patients. In this study, we analysed this respiration motion, investigated the effectiveness of an RPM-based respiration guiding system and determined suitable gating windows for RGRT of liver cancer.

## Materials and Methods

### Study overview

The retrospective data analysis of 52 liver patients with liver cancer enrolled in this study was approved by the Institutional Review Board of the Yeungnam University Medical Center (YUMC 2018-07-037). Patient consent was specially waived under the approval of the institutional review board given that patient anonymity was ensured. All the methods were carried out in accordance with the relevant guidelines and regulations.

### Patient data

We examined data from 52 patients with liver cancer who received respiratory training on an RPM system (version 1.7; Varian Medical Systems, Palo Alto, CA, USA) and a respiration guiding system for radiotherapy between August 2016 and February 2018. The patients’ characteristics and treatment parameters are summarized in Table 1. The average age of the patients was 65 years, ranging from 47 to 85 years. There were 11 (21.2%) female and 41 (78.8%) male patients. From the patients, 33 (63.5%) and 19 (36.5%) had received intensity-modulated radiotherapy and stereotactic body radiotherapy.

**Table 1:** Patients’ characteristics and treatment parameters (*n* = 52).

Parameter	Value
Number of patients	52
Median ages (range)	65 (47–85) years
***Gender***	
Female	11 (21.2%)
Male	41 (78.8%)
***Radiotherapy technique***	
Intensity-modulated radiotherapy	33 (63.5%)
Stereotactic body radiotherapy	19 (36.5%)
***Volumes***	
GTV	68.1 (0.8–1322.7) cm^3^
PTV	105.0 (6.7–1479.8) cm^3^
Normal liver	1288.0 (807.0–2054.3) cm^3^
***Diagnosis***	
HCC	44 (84.6%)
CCC	1 (1.9%)
Metastasis	7 (13.5%)

GTV, gross target volume; PTV, planning target volume; HCC, hepatocellular carcinoma; CCC, cholangiocellular carcinoma.

### Respiratory training and respiration signal analysis

**Figure 1:**
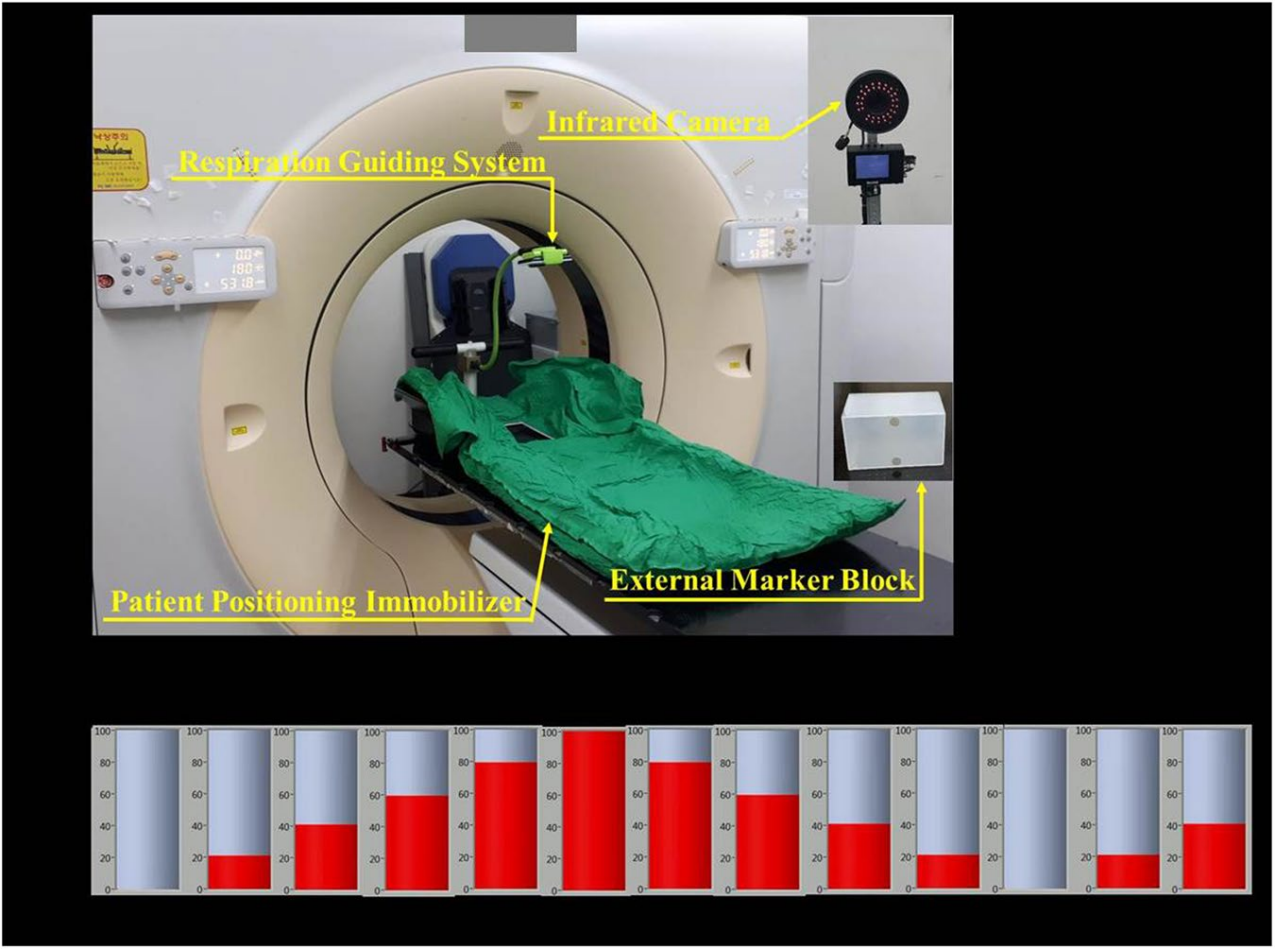
(**a**) Photograph of RPM and respiration guiding system installed in CT simulator room. (**b**) Schematic diagram of respiration guiding system with cycles of 3 s.

Figure 1a shows the setup for a patient to receive respiratory training on RPM and respiration guiding. Figure 1b shows a schematic diagram of the operation of the respiration guiding system for 3 s, which is a respiration cycle that patients with liver cancer should adopt according to the protocol of the Yeungnam University Medical Center. The respiration signals of the 52 liver cancer patients who underwent radiotherapy were recorded during 5 min of unguided followed by 5 min of guided respiration. Then, 4D CT was acquired in a CT simulation room for subsequent treatment. Details about respiratory training for patients and data extraction from the respiration signals are provided in our previous report^7^, which was focused on lung cancer. Unlike that work, we analysed the respiratory signals of patients with liver cancer in the present study. To exclude the required adaptation time of the patient to the respiratory guiding system, we analysed the respiration signal in 3–5 min from the 5-min guided respiration recording.

We applied a paired *t*-test using SPSS Statistics V.22 (IBM, Armonk, NY, USA) to determine the effectiveness of respiration training in the 52 liver patients, where *p* $\le$ 0.05 was considered as statistically significant. Furthermore, the standard deviation of the respiration in phase 30–70%, the volume of normal liver, the planning target volume (PTV), and gross target volume (GTV) were subjected to Pearson correlation analysis.

## Results

Supplementary Table 1 lists the results of the paired *t*-test with unguided and guided respiration using RPM and respiration guiding. During the analysed period, the patients performed from 16 to 39 respirations. Statistically significant differences are indicated with superscript ^*^. From the 52 patients, 47 exhibited significantly different unguided and guided respiration. The five patients (P#30, P#38, P#39, P#40, and P#41) who did not appear to benefit from respiration guiding were already capable of achieving stable 3-s respiration cycles prior training. Note that we limited the effect analysis to the respiration period of 3 s.

**Figure 2:**
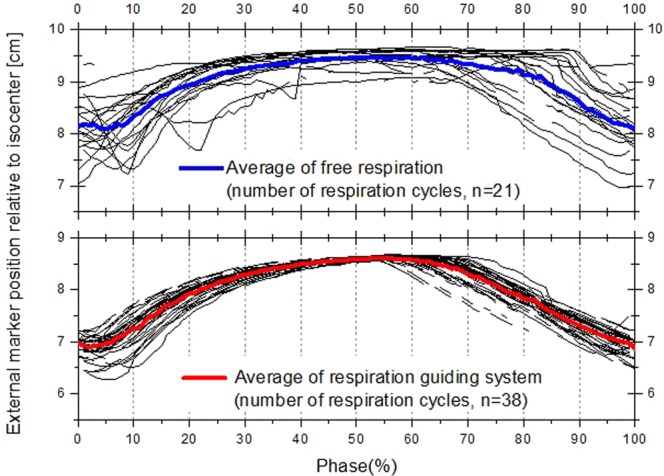
Respiratory signal periods according to phase for free respiration and with respiration guiding system for patient #28.

**Figure 3:**
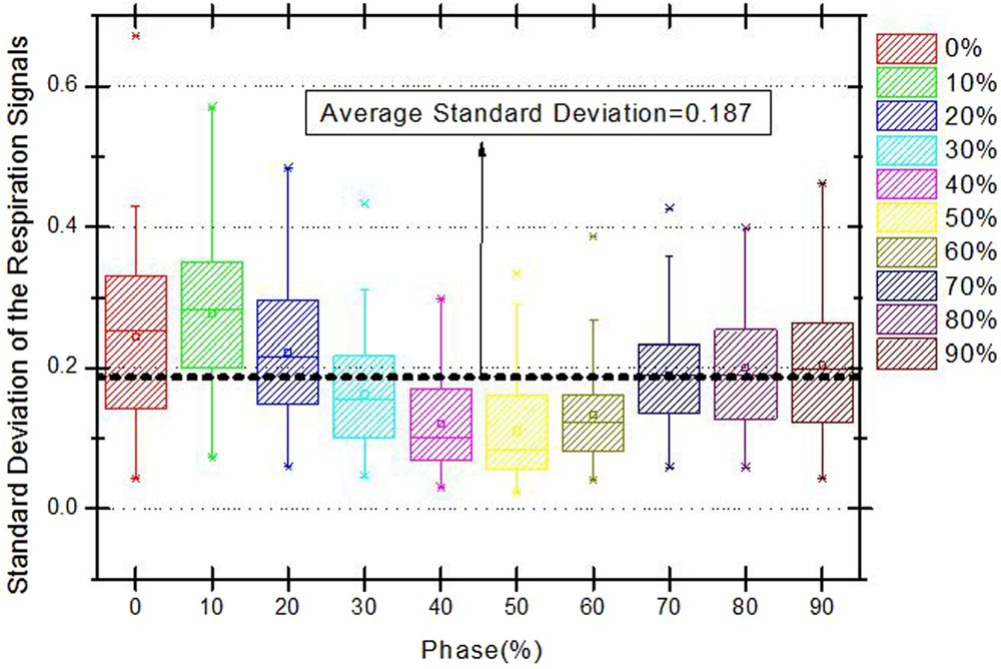
Standard-deviation box plot of guided respiratory signals according to respiration phase among all liver cancer patients (*n* = 52).

Figure 2 shows the overlaid respiratory signal periods of patient P#28 according to the respiratory phase (%) for both unguided (top graph) and guided (bottom graph) respiration. The respiratory signal, which is represented by the location of an external marker, using the guiding system is more stable and consistent than the unguided respiratory signal. Figure 3 shows the box plot of the standard deviation corresponding to the guided respiratory signals according to respiration phase obtained from the 52 liver cancer patients. The average standard deviation was 0.187, with the lowest variability occurring in respiration phase 30–60%.

**Figure 4:**
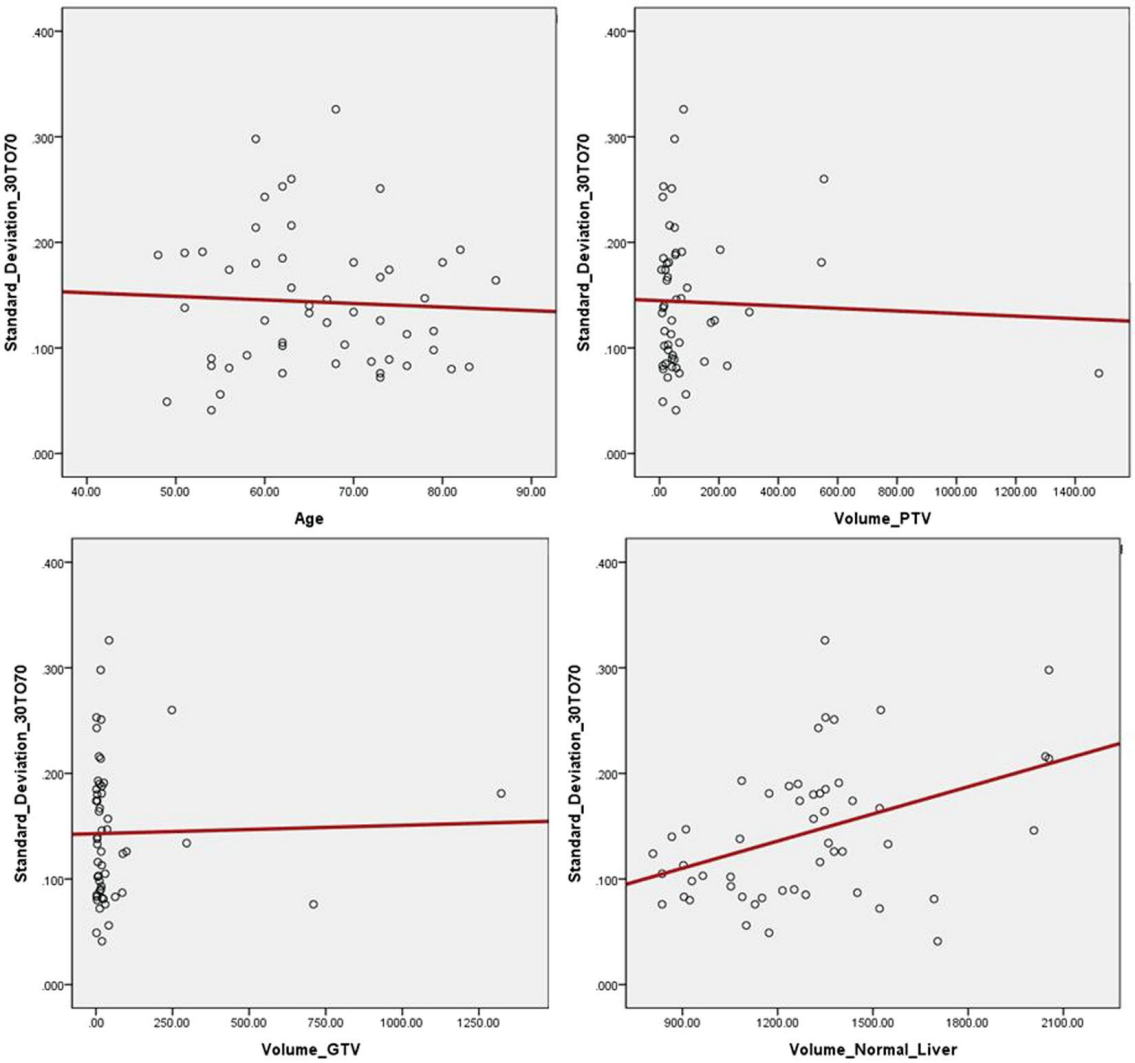
Scatter plot of standard deviation in the 30–70% respiration phase according to (**a**) age, (**b**) PTV, (**c**) GTV, and (**d**) volume of normal liver.

Overall, the Pearson correlation coefficient (*r*) is a linear relationship that can be considered negligible for values between −0.1 and 0.1, weakly positive between 0.1 and 0.3, strongly positive between 0.3 and 0.7, and very strongly positive between 0.7 and 1.0. In our study, the Pearson correlation between the standard deviation of respiration phase 30–70% and the volume of normal liver was 0.408, indicating a strong linear relationship. Figure 4 shows scatter plots relating the standard deviation in respiration phase 30–70% to age, PTV, GTV, and volume of normal liver. No linear correlation between the standard deviation and any of these parameters was found.

Table 2 lists the standard deviations (mean and range) of the respiratory signals from the 52 patients when using the respiration guiding system. Respiration phase 50% exhibits the lowest standard deviation, whereas phases 30–60% and 30–70% also have a relatively low variability.

**Table 2:** Standard deviations of respiratory signals from the 52 patients with liver cancer performing guided respiration.

Phase	Mean (range)
0%	0.244 (0.043–0.671)
10%	0.277 (0.072–0.571)
20%	0.222 (0.060–0.484)
30%	0.163 (0.046–0.433)
40%	0.120 (0.031–0.298)
50%	0.111 (0.024–0.334)
60%	0.133 (0.040–0.386)
70%	0.190 (0.059–0.426)
80%	0.200 (0.059–0.399)
90%	0.204 (0.043–0.462)

## Discussion

We evaluated the data from 52 liver cancer patients diagnosed with hepatocellular carcinoma, cholangiocellular carcinoma, and metastasis. The patients were trained in RPM and a respiration guiding system to receive RGRT. The respiratory signals with and without guidance were analysed to determine the most suitable gating window during radiotherapy. Although the study provided useful insights on the effectiveness of the respiration guiding system and the best gating window, some limitations remain to be addressed. For instance, we only considered the anterior–posterior direction for the external marker block and assumed a complete correlation between this and the superior–inferior direction. However, respiration-related motion of tumours can be very complex and varied along the anterior–posterior, lateral, and superior–inferior directions and the corresponding rotation axes^21,22^. Moreover, if the frequency or shape of the respiratory guiding system changes, the most suitable gating window can also change. Nevertheless, the selected frequency and shape in this study are commonly used in clinical practice.

Jiang *et al*.^23^ explained typical non-gated 3D conformal radiotherapy with duty cycle of 100% and gated 3D conformal radiotherapy with gating window of 30–50%. For step-and-shoot intensity-modulated RGRT, the duty cycle may be below 30% given the time required for the leaf motion of multi-leaf collimators.

Vedam *et al*.^24^ analysed two cases of patients with non-small cell lung cancer considering either the respiration amplitude or phase. The range of motion increased with the duty cycle in both the phase and amplitude modes. Hence, the authors suggested that the clinical target volume to PTV margins should increase for duty cycles from 40% along the cranio-caudal direction.

We have previously analysed the effect of respiration training using a visible guiding system in 23 lung cancer patients^7^. From them, 19 patients exhibited a significant difference between unguided and guided respiration. In addition, when the best gating window is limited by the reproducibility of the external marker position relative to the isocentre, we found the most suitable gating window for treatment of lung cancer to be between 30% and 70%.

In the present study, we examined the use of RPM and respiration guiding for improving RGRT of 52 liver cancer patients. We found that 47 of the 52 patients exhibited significantly improved reproducibility of the external marker position when using the respiration guiding system. The overall standard deviation of the respiration signal for duty cycle of 40% retrieved a suitable gating window in respiration phase 30–60%, whereas for duty cycle of 50%, a suitable gating window was in respiration phase 30–70%. Moreover, the Pearson correlation between the standard deviation of phase 30–70% and the volume of normal liver indicated that liver cancer with smaller relative volume may provide more reproducible respiration outcomes for applying RGRT.

## Conclusion

Among 52 liver cancer patients receiving RGRT and trained in RPM and respiration guiding, 47 exhibited significantly improved respiration reproducibility from unguided to guided respiration. Considering the lowest standard deviations of respiration signals among all patients, the most suitable gating window for RGRT of liver cancer corresponds to respiration phases of 30–60% or 30–70%.

## Supplementary information

Supplementary table
