# Supplementary material for: Optimal Gating Window for Respiratory-Gated Radiotherapy with Real-Time Position Management and Respiration Guiding System for Liver Cancer Treatment
Source: Sci Rep. 2019 Mar 13;9:4384. doi: 10.1038/s41598-019-40858-2 (PMC6416406; doi:10.1038/s41598-019-40858-2)
Supplement: Supplementary file 1 — Supplementary table [file 41598_2019_40858_MOESM1_ESM.pdf]

# Optimal Gating Window for Respiratory-Gated Radiotherapy with Real-Time Position Management and Respiration Guiding System for Liver Cancer Treatment

Se An Oh[1], Ji Woon Yea[1,2], Sung Kyu Kim[1,2], and Jae Won Park[1,2*]

[1]Department of Radiation Oncology, Yeungnam University Medical Center, Daegu, Korea

[2]Department of Radiation Oncology, Yeungnam University College of Medicine, Daegu, Korea

* Corresponding author:

E-mail: kapicap@ynu.ac.kr

# Supplementary Table Legends

Supplementary Table S1. Results of paired *t*-test between unguided and guided respiration signals with RPM and respiration guiding system ($n = 52$).

Supplementary Table S1.

| Patient | Respiration | Mean±SD (s) | Difference between paired data | | |
|---|---|---|---|---|---|
| | | | Number of respiration signals | Mean±SD (s) | *p*-value of paired *t*-test |
| P#1 | Free | 4.483±0.936 | 26 | 1.525±0.947 | <0.001* |
| | Guided | 2.958±0.161 | | | |
| P#2 | Free | 3.865±0.715 | 29 | 0.606±1.095 | 0.006* |
| | Guided | 3.259±0.836 | | | |
| P#3 | Free | 4.674±0.404 | 24 | 1.709±0.414 | <0.001* |
| | Guided | 2.966±0.101 | | | |
| P#4 | Free | 3.523±0.265 | 33 | 0.558±0.316 | <0.001* |
| | Guided | 2.964±0.159 | | | |
| P#5 | Free | 6.732±2.770 | 17 | 3.759±2.751 | <0.001* |
| | Guided | 2.974±0.212 | | | |
| P#6 | Free | 4.673±0.948 | 25 | 1.475±1.236 | <0.001* |
| | Guided | 3.198±0.958 | | | |
| P#7 | Free | 5.018±0.989 | 23 | 2.057±1.025 | <0.001* |
| | Guided | 2.961±0.235 | | | |
| P#8 | Free | 3.623±1.369 | 32 | 0.656±1.372 | 0.011* |
| | Guided | 2.967±0.197 | | | |
| P#9 | Free | 3.730±0.480 | 31 | 0.758±0.507 | <0.001* |
| | Guided | 2.972±0.170 | | | |
| P#10 | Free | 3.429±0.743 | 34 | 0.463±0.770 | 0.001* |
| | Guided | 2.966±0.257 | | | |

| | | | | | |
|---|---|---|---|---|---|
| P#11 | Free | 5.574±1.258 | 20 | 2.447±1.474 | <0.001* |
| | Guided | 3.126±0.449 | | | |
| P#12 | Free | 4.677±1.161 | 24 | 1.588±1.357 | <0.001* |
| | Guided | 3.089±0.655 | | | |
| P#13 | Free | 5.043±0.756 | 23 | 2.073±0.737 | <0.001* |
| | Guided | 2.970±0.225 | | | |
| P#14 | Free | 3.917±0.495 | 29 | 0.956±0.577 | <0.001* |
| | Guided | 2.961±0.237 | | | |
| P#15 | Free | 3.359±0.380 | 34 | 0.382±0.403 | <0.001* |
| | Guided | 2.978±0.163 | | | |
| P#16 | Free | 4.251±1.832 | 27 | 1.287±1.873 | 0.001* |
| | Guided | 2.965±0.257 | | | |
| P#17 | Free | 6.536±1.193 | 18 | 3.401±1.459 | <0.001* |
| | Guided | 3.135±0.740 | | | |
| P#18 | Free | 3.925±0.669 | 30 | 3.401±1.459 | <0.001* |
| | Guided | 2.959±0.325 | | | |
| P#19 | Free | 3.401±0.561 | 34 | 0.333±0.801 | 0.021* |
| | Guided | 3.069±0.545 | | | |
| P#20 | Free | 5.289±0.952 | 22 | 2.321±0.945 | <0.001* |
| | Guided | 2.968±0.213 | | | |
| P#21 | Free | 3.264±0.310 | 36 | 0.297±0.376 | <0.001* |
| | Guided | 2.967±0.202 | | | |
| P#22 | Free | 3.273±0.336 | 36 | 0.294±0.406 | <0.001* |
| | Guided | 2.978±0.208 | | | |
| P#23 | Free | 4.144±0.643 | 27 | 1.155±0.808 | <0.001* |
| | Guided | 2.988±0.451 | | | |

| | | | | | |
|---|---|---|---|---|---|
| P#24 | Free | 4.124±1.811 | 27 | 1.154±1.772 | 0.002* |
| | Guided | 2.970±0.178 | | | |
| P#25 | Free | 5.409±0.566 | 21 | 2.450±0.478 | <0.001* |
| | Guided | 2.959±0.232 | | | |
| P#26 | Free | 5.431±0.962 | 21 | 2.452±1.045 | <0.001* |
| | Guided | 2.979±0.260 | | | |
| P#27 | Free | 3.509±0.337 | 33 | 0.554±0.459 | <0.001* |
| | Guided | 2.955±0.462 | | | |
| P#28 | Free | 5.092±1.133 | 21 | 2.119±1.216 | <0.001* |
| | Guided | 2.973±0.191 | | | |
| P#29 | Free | 4.501±0.714 | 25 | 1.533±0.792 | <0.001* |
| | Guided | 2.967±0.164 | | | |
| P#30 | Free | 2.826±0.934 | 39 | -0.138±1.079 | 0.430 |
| | Guided | 2.964±0.404 | | | |
| P#31 | Free | 3.929±0.261 | 29 | 0.725±0.606 | <0.001* |
| | Guided | 3.204±0.542 | | | |
| P#32 | Free | 4.762±0.494 | 24 | 1.795±0.516 | <0.001* |
| | Guided | 2.967±0.183 | | | |
| P#33 | Free | 5.474±2.004 | 21 | 2.501±2.013 | <0.001* |
| | Guided | 2.973±0.213 | | | |
| P#34 | Free | 6.440±3.133 | 16 | 3.502±3.241 | 0.001* |
| | Guided | 2.938±0.217 | | | |
| P#35 | Free | 2.856±0.876 | 38 | -0.189±1.063 | 0.281 |
| | Guided | 3.045±0.568 | | | |
| P#36 | Free | 4.646±0.591 | 25 | 1.680±0.643 | <0.001* |
| | Guided | 2.966±0.306 | | | |

| | | | | | |
|---|---|---|---|---|---|
| P#37 | Free | 2.983±0.316 | 39 | 0.271±0.360 | <0.001* |
| | Guided | 2.712±0.175 | | | |
| P#38 | Free | 3.351±0.539 | 34 | 0.455±0.673 | <0.001* |
| | Guided | 2.896±0.286 | | | |
| P#39 | Free | 2.746±0.375 | 39 | -0.144±0.586 | 0.134 |
| | Guided | 2.890±0.407 | | | |
| P#40 | Free | 3.188±0.420 | 36 | 0.083±0.644 | 0.446 |
| | Guided | 3.105±0.479 | | | |
| P#41 | Free | 3.202±0.535 | 36 | 0.106±0.639 | 0.328 |
| | Guided | 3.097±0.380 | | | |
| P#42 | Free | 3.601±0.319 | 32 | 0.533±0.598 | <0.001* |
| | Guided | 3.069±0.472 | | | |
| P#43 | Free | 4.975±0.592 | 23 | 2.021±0.547 | <0.001* |
| | Guided | 2.954±0.276 | | | |
| P#44 | Free | 4.719±2.634 | 24 | 1.578±2.744 | 0.010* |
| | Guided | 3.141±0.447 | | | |
| P#45 | Free | 3.259±0.213 | 35 | 0.295±0.241 | <0.001* |
| | Guided | 2.964±0.161 | | | |
| P#46 | Free | 4.422 | 26 | 1.446±0.701 | <0.001* |
| | Guided | 2.976 | | | |
| P#47 | Free | 3.490 | 33 | 0.529±0.905 | 0.002* |
| | Guided | 2.960 | | | |
| P#48 | Free | 6.317 | 18 | 3.339±3.476 | 0.001* |
| | Guided | 2.979 | | | |
| P#49 | Free | 4.642 | 25 | 1.544±0.789 | <0.001* |
| | Guided | 3.098 | | | |

| | | | | | |
|---|---|---|---|---|---|
| P#50 | Free | 4.256 | 27 | 1.237±0.939 | <0.001* |
| | Guided | 3.019 | | | |
| P#51 | Free | 4.963 | 23 | 1.986±2.225 | <0.001* |
| | Guided | 2.977 | | | |
| P#52 | Free | 3.479 | 33 | 0.513±0.677 | <0.001* |
| | Guided | 2.967 | | | |
